# A conversation with Nora Volkow

**DOI:** 10.1172/JCI157462

**Published:** 2022-02-01

**Authors:** Ushma S. Neill

Through trailblazing imaging studies of the brain’s frontal cortex and its dopamine-driven circuitry, Nora Volkow, director of the National Institute on Drug Abuse (NIDA), has helped to reveal the neurobiological underpinnings of addiction and how drug-induced changes in brain chemistry contribute to its trademark craving, compulsion, and loss of control. To hear more of Volkow’s (Figure 1) views on the value of being an effective communicator and lessons learned from the double pandemic of opioids and COVID-19, see the full video on the *JCI* website (https://www.jci.org/videos/cgms).

*JCI*: What were you like as a child?

Volkow: I was born in Mexico City. My father, who was born in Russia, is the grandson of Leon Trotsky. My three sisters and I grew up in the house where Trotsky was killed; it’s now a museum, but at the time it gave us an extraordinary opportunity to explore and to be part of an event in history that was very consequential. My mother was born in Spain and came to Mexico because of the civil war in Spain, when Mexico gave political asylum to the children of those who were fighting against Franco. I was born with this background of two major civil wars: the Russian Revolution and the Spanish civil war. That imprinted me early on that we are all part of something that’s much more than just your life at that moment; there is a continuity of what you do that will affect the next generations.

My father is a chemist, and he had his laboratory in the house itself. That allowed me to make science an everyday life event. My mother was a successful clothing designer, with an extremely engraved sense of aesthetics. I had on the one hand, my mother’s aesthetic artistic component, and on the other, my father’s very scientific rigorous thinking process. As I look at things retrospectively, I realize that I grew up in a culture where the work of males was considered more important than that of females, which I am certain influenced me towards trying to emulate more what my father than what my mother was doing.

I loved school. I am someone that has always loved learning. I also loved being in nature and enjoyed my father taking us hiking in the mountains or rural areas in Mexico. When I recall growing up as a child, what is very clear is that I was fascinated by other humans. I could spend hours watching people interacting with one another. This is also why I enjoyed reading so much, because it allowed me to get into someone else’s brain and life perceptions, which is where my curiosity has always resided: what would it feel to be another person, and how would I comprehend things differently?

*JCI*: Is it this fascination with understanding people that pushed you towards medical school instead of the scientist route?

Volkow: I don’t know of any other profession where you are in front of someone that is, in a metaphorical sense, naked. That is, without posturing but just being themselves. It has always been very appealing to me to have human interactions that are genuine and to be able to help someone in a meaningful way.

When I finished medical school, I was going to do a PhD to have both experiences. In the end, I opted to do a residency in psychiatry because of the human experience it gave me to interact with people who — because of a brain disorder — have a disruption of how they perceive and interact with the world. In many instances, it is helpful to investigate what are the behavioral/clinical consequences of the disruption of the neuronal system affected by the disease. I was particularly fascinated by addiction, but I was also engrossed by psychosis and schizophrenia.

*JCI*: What was the nature of the research you did in medical school?

Volkow: Pharmacology. I was attracted to studying the drugs that can produce addiction. Here you have a chemical that can take over the behaviors and preferences of an individual. Being very attuned to a person’s right for freedom and free choice, I wanted to understand how a drug could potentially remove the capacity of a person for self-determination. How does a drug hijack the neurocircuitry that drives motivation and drives behaviors that are so devastating to the addicted person? I first started working with cocaine because cocaine is among the most addictive of drugs.

*JCI*: At that time, were you planning to be a medical doctor focused on patients?

Volkow: I wanted to do clinical research, and that’s why I went into imaging, because it provides the tool that allows direct investigation of the human brain. When I was a medical student, what we knew about how the brain works was based on postmortem studies of people who died of stroke; you could link the location of the stroke with the behavioral deficits or by observing the behavioral consequences to patients from localized seizures or brain tumors. As I was finishing medical school, the first paper came out on the use of positron emission tomography (PET) to investigate the function of the human brain. After reading the paper, I told my father, “I’d like to go to New York to learn about this,” and I asked him if he would support me with the ticket and lodging costs. He did not hesitate and that is how I started.

*JCI*: You mentioned before, you had been admitted for graduate school- to MIT — but you did not attend?

Volkow: I had seven months before I needed to start my PhD at MIT. My intent in going to NY was to meet the chairman of the department of psychiatry at NYU (Dr. Cancro) where the brain imaging studies were being done. Against all odds, since he did not know me, he met with me and arranged for me to volunteer with the brain-imaging studies. He liked my work and asked me to consider doing residency training in psychiatry that would allow me to continue to do the brain-imaging work I had embarked on. It was easy to convince me; I think I immediately agreed. However, it was hard for me to tell the director of the MIT neuropsychology program, who was going to be my mentor, that my plans had changed.

*JCI*: The PET imaging you were able to do at NYU was focused on schizophrenia.

Volkow: Schizophrenia is a disorder that has always fascinated me. It’s related to the notion that we have a consensus of what reality is, which we process automatically amidst the complex organization and function of the human brain. I wanted to understand, how do we comprehend reality? How do we know that a voice is real as opposed to imaginary? This required a means to look inside a functioning brain. Postmortem studies done on schizophrenia patients did not reveal any structural defects, which was different from the brains of those who died of a stroke or with epilepsy.

*JCI*: You had some momentum, but your residency finished. What drew you to Houston and the University of Texas to continue your work on brain imaging?

Volkow: What drew me to Houston, Texas, was their state-of-the-art imaging capabilities. It was the largest imaging laboratory that I had even seen. It was just magnificent. And they were also involved in the development of PET cameras themselves. I wanted to continue my work with schizophrenia, but rapidly realized that in the hospital where I was working, there were no patients being admitted with a diagnosis of schizophrenia. On the other hand, at that time, there was a rising problem with cocaine, and we were seeing patients arriving to the emergency department with psychotic episodes triggered by cocaine.

I figured if I could not study schizophrenia, then I could study cocaine-induced psychoses. So my brain-imaging studies with drugs were not initially focused on addiction, but on psychotogenic actions and toxicity.

*JCI*: Nobody really believed you at the beginning — that drugs could induce changes in the brain.

Volkow: You do science and science surprises you. I started to do imaging of people that were using cocaine. We were measuring cerebral blood flow using PET, with ^15^O water and brain glucose metabolism with ^18^F-FDG. From the very first studies, what was most dramatic was how profoundly disrupted cerebral blood flow was in patients who were using high doses of cocaine regularly. Their brains looked like the brains of patients who had a stroke. Upon seeing one of my patient’s brain scans, one of the cardiologists commented, “That looks like a stroke in the brain.” That patient was not an exception.

There were two things that were notable from our brain findings: how frequent brain blood flow defects were and how severe. But also intriguing to me was that even though patients had a marked reduction in cerebral blood flow, akin to that seen in patients with strokes who presented with paralysis, aphasia, or other symptoms, we were not seeing this in the cocaine-using patients. There was a discrepancy between what the brain images were showing and the minimal clinical evidence of cerebrovascular pathology. Follow-up evaluation of these patients with brain glucose metabolism imaging also showed reductions in glucose metabolism in the areas that were most affected by loss of blood flow, but the magnitude of the changes was much smaller.

It’s now well understood that cocaine triggers long-lasting vasoconstriction of blood vessels in the brain, decreasing cerebral blood flow, which would explain our findings. However, it is also possible, based on what we now know, that severe reductions in brain uptake of ^15^O water might have also reflected impaired transfer of water from blood vessels into the brain, an effect that could have implications for the glymphatic system.

People did not believe that cocaine was harmful. The *New England Journal of Medicine* rejected the paper on the basis that we had no evidence that cocaine was harmful, since patients did not present with neurological deficits. I also submitted a grant to NIDA, and they rejected it based on lack of evidence of neuropathology from cocaine.

I was unable to get the paper published until reports of individuals suffering from strokes and myocardial infarctions after cocaine use started to appear in the media. It became clear that cocaine was not a safe drug and that one of the negative effects was related to cerebrovascular pathology. These effects of cocaine are now very well recognized. However, at the beginning, like with anything else that overturns dogma and particularly for findings that rely on a new technology, it was very difficult to convince others. Another element that did not help me at that time was that I was a woman with an Hispanic accent. But regardless, if you persevere and the data are correct, it will eventually get integrated.

*JCI*: Why leave that imaging Shangri-la in Houston to come to Brookhaven National Laboratory?

Volkow: Brookhaven National Laboratory (BNL) had the imaging capability coupled with an extraordinary radiochemistry laboratory. I was ambivalent about moving to BNL, but when the director said, “Can we convince you if we label cocaine with carbon 11?” that was a very tempting offer, as ^11^C is a positron emitter and to use it to label cocaine would allow me to do things beyond imagination. It would give me the ability to use PET to measure where cocaine went in the brain and assess its pharmacokinetics in vivo in the human brain.

Subsequently, one of my BNL colleagues developed a method that allowed him to measure changes in dopamine with PET in nonhuman primates. I took this methodology and applied it to study humans, which allowed me for the first time ever to use PET to measure the increases in dopamine produced by a drug directly in humans while in parallel measuring the associated behavioral changes. I then expanded the use of this very powerful tool to understand the involvement of dopamine in addiction in humans and its role in motivating our choices and actions.

*JCI*: In the early 2000s, you transitioned to be the director of NIDA. How was the then–NIH director, Zerhouni, able to entice you?

Volkow: Zerhouni convinced me that the impact that I had as a scientist was much more restricted than what I could have as a director of NIDA. I had been obsessed since I was a medical student with how the health-care system neglects addiction, treating it as a defect of character. It was also very clear to me how deleterious and detrimental the failure of health care to screen and treat addiction was to patients. The position of NIDA director would give me the opportunity to help change this.

So as NIDA director, this became one of my priorities. It has been challenging, for it’s much easier for people to accept a cardiac or a metabolic condition as a disease than it is to accept addiction, which involves our actions, as a disease. Stigma and lack of training continue to be a major obstacle among clinicians, making them ineffectual in their ability to screen or treat addiction.

The pernicious consequence of this neglect was made clearly evident by the opioid crisis that is raging in our country, for the lack of training by clinicians in addiction was one of its main culprits. Clinicians were prescribing very potent and addictive drugs with no understanding about their effects and no ability to recognize those at higher risk for addiction nor detect when their patients were becoming addicted.

*JCI*: If you could not have been a physician or a scientist, what career path do you think could have kept you as motivated?

Volkow: A career in art, as art uses the language of emotions to give us a different means to experience and understand our world and to share it with others.

## Figures and Tables

**Figure 1:**
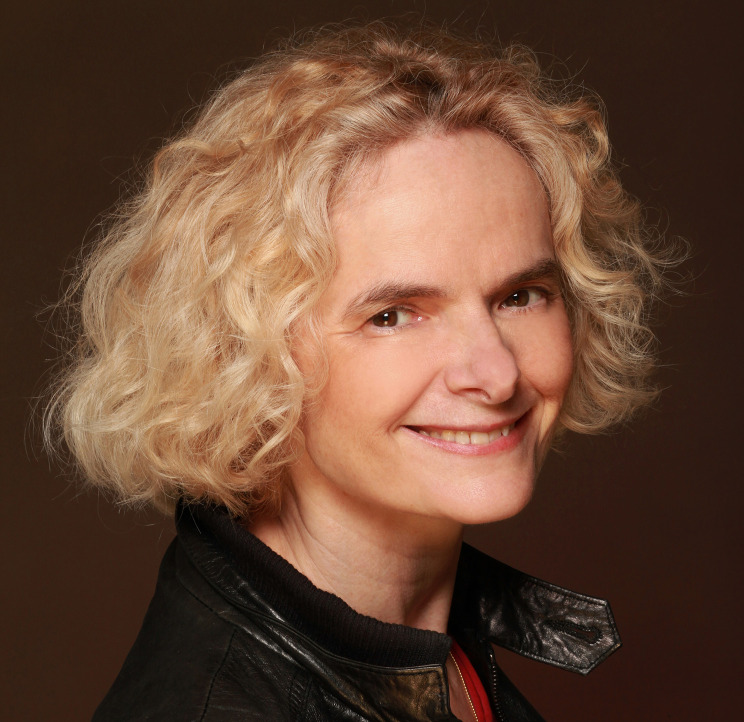
Nora Volkow. Image credit: Mary Nobel Ours.

## Version 1 02/01/2022

Electronic publication

